# A Mobile App (iBeni) With a Neuropsychological Basis for Cognitive Stimulation for Elderly Adults: Pilot and Validation Study

**DOI:** 10.2196/resprot.9603

**Published:** 2018-08-21

**Authors:** Claudia I Martínez-Alcalá, Alejandra Rosales-Lagarde, Esmeralda Hernández-Alonso, Roberto Melchor-Agustin, Erika E Rodriguez-Torres, Benjamín A Itzá-Ortiz

**Affiliations:** ^1^ Consejo Nacional de Ciencia y Tecnología Ciudad de México Mexico; ^2^ Department of Gerontology School of Health Sciences Universidad Autónoma del Estado de Hidalgo Pachuca Mexico; ^3^ Department of Information Technology Instituto Tecnológico Superior de Zacapoaxtla Puebla Mexico; ^4^ Centro de Investigación de Matematicas Universidad Autónoma del Estado de Hidalgo Mineral de la Reforma, Hidalgo Mexico

**Keywords:** mobile application, cognitive stimulation, cognitive impairment, older adults, neuropsychological evaluation.

## Abstract

**Background:**

Cognitive impairment is considered one of the most feared chronic conditions among the older adult population since its incidence is approximately twice more frequent than that of dementia. In Mexico, no studies or reports of older adults using technology for cognitive interventions have been published, given that institutions usually frame cognitive stimulation tasks in paper and pencil (ie, in the traditional manner).

**Objective:**

The objective of this study was to create and analyze the effect, viability, and impact of a mobile app for cognitive stimulation implemented among a group of elderly adults (over 60 years of age) from the state of Hidalgo in Mexico.

**Methods:**

This study was a nonprobabilistic pilot trial using convenience sampling. An intervention was implemented among a group of 22 older adults between 60 and 80 years of age over 12 weeks. Half of the older adults were stimulated with the mobile app (experimental group) and the other half followed the traditional paper and pencil training (control group). Assessments with the Mini-Mental State Examination (MMSE) and the Neuropsi, a neuropsychological test validated in Mexico, were done before and after both cognitive stimulations.

**Results:**

According to the analyzed data, 6/11 (55%) participants from the experimental group obtained better results in their cognitive skills, and 5 (45%) of the adults maintained their score, given that the participants were able to execute the exercises repetitively. Meanwhile, for the control group, only 3/11 (27%) participants obtained better results in the postevaluation. Significant values for results of the MMSE were obtained in the postevaluation for the experimental group compared to the control group, while results did not show significant differences in the Neuropsi. Regarding the validation of the app, all the participants evaluated its pertinence positively.

**Conclusions:**

The intervention data show that the experimental group obtained better results in the postevaluation given that the participants were able to execute the exercises repetitively. The control group could not accomplish this since they had to respond on the manual and no further attempts were provided. However, both groups increased their score in the neuropsychological evaluations. This suggests that a longer and more frequent intervention is required.

**Registered Report Identifier:**

RR1-10.2196/9603

## Introduction

The prevalence of chronic diseases related to aging is becoming a significant public health issue as the rate of the elderly population increases worldwide [1-3], particularly in Mexico [4]. Cognitive impairment is a chronic condition that is considered one of the most feared by the older adult population since its incidence is approximately twice as frequent compared to dementia [5]. According to projections, it is estimated that by 2050 the number of adults over the age of 60 will be around two billion and it will represent 22% of the world population. Similarly, four-fifths of this older adult population will live in developing countries (ie, Africa, Asia, and Latin America) [6-8].

Consequently, several studies have evaluated the effectiveness of different cognitive interventions on cognitively preserved older adults and populations with mild cognitive impairment (MCI) [9-13]. One of the key findings of these studies is that cognitive ability is significantly preserved with the use of these interventions, which are mainly focused on memory and function. As a result, institutions are recommending their implementation as a preventive step.

The terms *cognitive stimulation* and *cognitive training* have been used interchangeably. However, these terms do not necessarily mean the same thing, so it is crucial to describe, define, and clarify these 2 intervention methods. Bigand and Tillman [14] mention that cognitive stimulation involves actions directed to maintaining or improving cognitive skills and is based on the pyschopedagogical planning of activities aimed at activating and maintaining mental skills. For their part, Li et al [15] define cognitive training as the teaching of strategies and theoretical abilities that allow the enhancement of cognitive functions, particularly with the intention of improving specific domains.

Thus, it is possible to establish that cognitive stimulation aims at maintaining and preserving cognitive functions in individuals that are relatively healthy or have a MCI, boosting their preserved cognitive capacity and skills, and decelerating cognitive decline process. Moreover, this intervention can be applied to any individual since anyone can improve his or her cognitive capacities to be more cognitively skillful.

Information and Communication Technologies (ICT) have assisted cognitive stimulation. These tools have increased and expanded recently due to greater availability of mobile devices (ie, mobile phones and tablets) in everyday life [16]. Health professionals and researchers have adopted these devices as an alternative intervention when dementia is presented or when the intention is to preserve the patient’s cognitive capacity, even in patients with mild cognitive decline [17-20].

Studies have affirmed that one of the strengths of this type of intervention is that they offer instant feedback. By using ICTs, they can adapt the tasks dynamically to the particular needs of the patient, even about their progress in the completion of tasks [21-22]. Also, Martinez-Alcala et al [23] indicate that technologically assisted interventions involve structured task practice and cognitively challenging exercises and that they offer several advantages over traditional methods, including visually attractive interfaces, efficient result delivery and the ability to adapt to the patient’s progress.

Likewise, some studies have demonstrated that cognitive improvement does not transfer easily to the realization of new tasks and that these yield better results when established repetitively with increasing levels of difficulty [24]. Similarly, other studies have shown that cognitive stimulation interventions can be administered not only through therapist instruction but also through computerized technology [25-26].

Recently, interest has thus been focused on interventions based on computerized cognitive stimulation or technologically-assisted cognitive stimulation. Regarding cognitive impact, this is justified because interventions supported by ICT can have a positive impact on attention measures, executive functions, and memory [11,17,20-22,24-26].

Based on a review of 226 cognitive interventions of ICT in healthy older adults and people with MCI between January 2015 and January 2018 in Pubmed, a total of 9 studies were included. Articles that did not match the corresponding period were excluded, as were the research studies that did not include people over 60 years of age. Those included had to be either healthy individuals or experiencing MCI, aimed at cognitive aspects, and written in English. From the 9 studies, 5 (56%) were about cognitive training, 2 (22%) used cognitive stimulation, and 2 were cognitive assessment studies (see Multimedia Appendix 1).

The findings from the recent research studies indicate that mobile phone apps can help healthy elderly people and with MCI in the enhancement of their quality of life by targeting these apps at their cognitive deficiencies such as memory loss [27-31]. Also, as the evidence shows, mobile phone apps can uniquely contribute to early diagnosis and assessment of dementia [27,32]. Generally, when people present subjective memory complaints and MCI, mobile phone apps can help them to be more independent and socially engaged.

Lu et al [33] argue that apps must involve end-users in the co-design of new technologies to develop tailored devices, as well as testing them in a real-world context. The results showed that the cognitive training game developed in this study was accepted by the nine participants included in the study, and a high degree of satisfaction was noted. Yasini and Marchand [28] indicate that the use of tablets and the development of serious games in close cooperation with health professionals and elderly patients (ie, the end user) are likely to provide satisfactory results to improve health care for those patients suffering from cognitive disorders.

Furthermore, Zygouris et al [32] and Zorluoglu et al [27] show evidence that mobile phone apps are effective in cognitive screening and the assessment and diagnosis of MCI. One advantage of apps is that they are more accurate than traditional manual testing. They are easily administered and understood by the elderly. They also save time, minimize the examiner's biases, can provide an early diagnosis enabling patients to stay independent on their tasks of daily living, they may cut hospitalization and treatment costs, they can improve the overall quality of life of the elderly, and they are ecological [27-35].

Considering the studies mentioned above, the cognitive interventions based on ICT can provide support for healthy seniors and MCI in the early diagnosis of this cognitive disorder to improve their cognitive functions. Also, they can reduce both the mental and economic burden of subjects and their caregivers.

It is important to point out that in Mexico only a few institutions aimed at the elderly are endowed with cognitive stimulation programs, and even these interventions lack pre- and postneuropsychological studies. Also, exercises in the training lack a neuropsychological basis, are extracted from books in an unsystematized way and are not entirely validated. In the same vein, no studies or reports on seniors using technology for these interventions have been published since institutions usually frame cognitive stimulation tasks in a traditional manner. Also, digital literacy levels among elderly Mexicans is very low. The population lacks access to technology and many feel incapable of learning how to use computers and the internet. According to the National Institute of Statistics and Geography at the Instituto Nacional de Estadistica y Geografia [36], only 2% (1/10) of Mexican seniors have access to technological devices. Due to the progressive nature of cognitive impairment, and the need for this type of intervention, a cognitive stimulation app aimed at the senior Mexican population was designed and implemented. The creation of this app reduces the need for human resources after a short period of training and adds efficiency.

This study describes a pilot nonrandomized study seeking to examine the effect, viability, and impact of the app. For this, an intervention was carried out with 22 adults 60-80 years of age. This population was divided into a control group, which executed cognitive stimulation exercises in a traditional manner (ie, paper and pencil), and an experimental group (EG), which used the app. Assessments with the Mini-Mental State Examination (MMSE) and the Neuropsi (a neuropsychological test validated in Mexico) were done before and after both cognitive stimulations. This study also shows that this technology and its benefits over traditional methods were validated.

## Methods

### Participants

This was a pilot nonrandomized and unblinded study where the participants self-allocated. For the recruitment of participants, a talk was held at the Centro Gerontológico Integral (CGI) at Punta Azul, Pachuca de Soto, where the objectives of the intervention were presented. Subsequently, all subjects were summoned through an advertisement offline. Those who accepted to attend the digital workshop at the Instituto de Ciencias de la Salud (ICSa), Universidad Autónoma del Estado de Hidalgo (UAEH) who had access to the app (explained below) represented the EG.

The study involved 22 seniors between 60-80 years of age. The EG participants underwent cognitive stimulation through an app. This group included 11 older adults (7 women and 4 men) with a mean of 64 years of age and had 6 years of academic study. It is important to point out that these adults attended a digital literacy course before implementation of the cognitive stimulation to develop the necessary technical competencies that enabled them to use the app more efficiently.

The control group (CG) included 11 older adults (8 women and 3 men) with a mean of 69 years of age and with 12 years of academic study. These adults did not have digital skills and did not participate in the digital workshop. They underwent cognitive stimulation in a traditional manner (ie, paper and pencil). It is important to note that both groups carried out the same exercises, with a difference only in the methods used. None of the participants discontinued the intervention.

Similarly, during the intervention, both groups were assisted by 2 students of gerontology and a project leader (CIMA). Even though the exercises for the EG were fully automated (ie, the app automatically increased the level according to the adult's progress) the study authors indicated the type of exercise they should perform and provided support to the participants at all times. For example, they provided encouragement so that any doubts of discontinuation would not arise during the use of the app. For their part in the CG, the authors followed each participant to complete the exercises in the manual.

The exclusion criterion adopted in the study for both groups was that no participant presented any untreated visual or hearing impairment. Table 1 shows the characteristics of both groups who participated in the study. As it can be seen, there were no significant differences between groups in age and education.

All participants were actively involved in the cultural and social activities at their nearest gerontology center. Also, it is important to mention that this study was reviewed and approved by the Research and Ethics Committee at the ICSa, UAEH. At the start of the intervention, participants provided written informed consent (see Multimedia Appendix 2). In the case of the EG, informed consent was provided electronically, and upon acceptance, registration was then carried out.

### Measures and Evaluations: Cognitive Evaluation

Before admittance to the program, participants undertook a neuropsychological evaluation through standardized tests called the Mini-Mental State Examination (MMSE) and the Neuropsi. The average time of administration was 40-60 minutes. The aim was to characterize the different aspects of the groups’ cognitive functions. The Neuropsi is a validated test in Mexico with norms according to age and educational level [37]. The maximum score on the test is 130. Neuropsi measures several neuropsychological functions: orientation, attention and concentration, verbal and visual-spatial memory, language, writing, reading, comprehension, conceptual executive functions, and motor executive functions. The evaluation also included other exploration tests based on the level of autonomy and ability to carry out basic Activities of Daily Living (ADL) or Barthel Scale. Furthermore, information on personal relative variables was gathered to know whether participants had a predisposition to cognitive impairment or any dementia. The variables included gender, academic level, and family background. Likewise, 2 questions of self-perception of their cognitive state were included. For both groups, face-to-face interventions were performed to ask about their name, age, and level of education. Also, both groups had face-to-face assessments of their neuropsychological level. Only the cognitive stimulation exercises for the EG were Web-based assessed. Finally, prepilot tests were carried out before compiling the final release of the app in which the seniors interacted with the app to know if the cognitive stimulation exercises had been correctly presented (eg, design and functionality) and the records from each participant were saved in the database. It should be mentioned that prepilot tests were performed on a group of 50 seniors from the IGC, but none participated in this study. These pretests provided some technical considerations for the app development and are described elsewhere in this manuscript.

### Description of the Mobile Application iBeni

The objective of this study was to create an app with a neuropsychological basis for elderly adults. This app was developed given the need for this type of intervention in Mexican communities. Likewise, this research was conducted as part of a CONACyT Commission at the UAEH through the Cátedras Program where the researchers (CIMA and ARL) are assigned.

The cognitive stimulation app primarily aims to improve cognitive functions and to decelerate the impairment process in healthy older adults or older adults with mild indicators of decline. The cognitive areas stimulated in the app were memory, attention, comprehension, perception, and visual-spatial processes. As previously mentioned, the exercises included in the app were specially designed for the elderly population. Moreover, each of the exercises were comprised of 3 levels of difficulty (low, medium, and high), which were selectively enabled as the users progressed.

The app had the advantage of cognitively stimulating seniors not only in memory but in other neuropsychological functions not found in other apps. It is important to notice that the exercises were based on items that appear on the MMSE and the Neuropsi tests. However, the app followed a graded level of difficulty based on the progress of the user. For example, in the first level of attention exercises, the user clicks (or selects) 1 image that does not coincide with the other. In subsequent levels, the app shows more objects but the user must select more images in less time. As previously mentioned, before compiling the final version of the app, prepilot tests were carried out to find out if the cognitive stimulation exercises were working correctly. These tests also confirmed that no change would be applied to both the content and the organization of the exercises included in the app.

#### Technical Considerations

Since the elderly population presents other age-related changes apart from cognitive decline, additional aspects were considered. These were (1) visual deterioration including a decrease in close focusing ability, contrast sensitivity decline and color differentiation alterations, (2) hearing loss, especially loss of the ability to detect high-pitched sounds, to decipher fast language and to understand speech in noisy environments; and (3) psychomotor deficiencies, including delayed responses in complex psychomotor tasks, a decrease in the ability to track moving targets, and low accuracy in fine movements. Consequently, the authors considered the following guidelines for the design of the app (Textbox 1).

**Figure 1:**
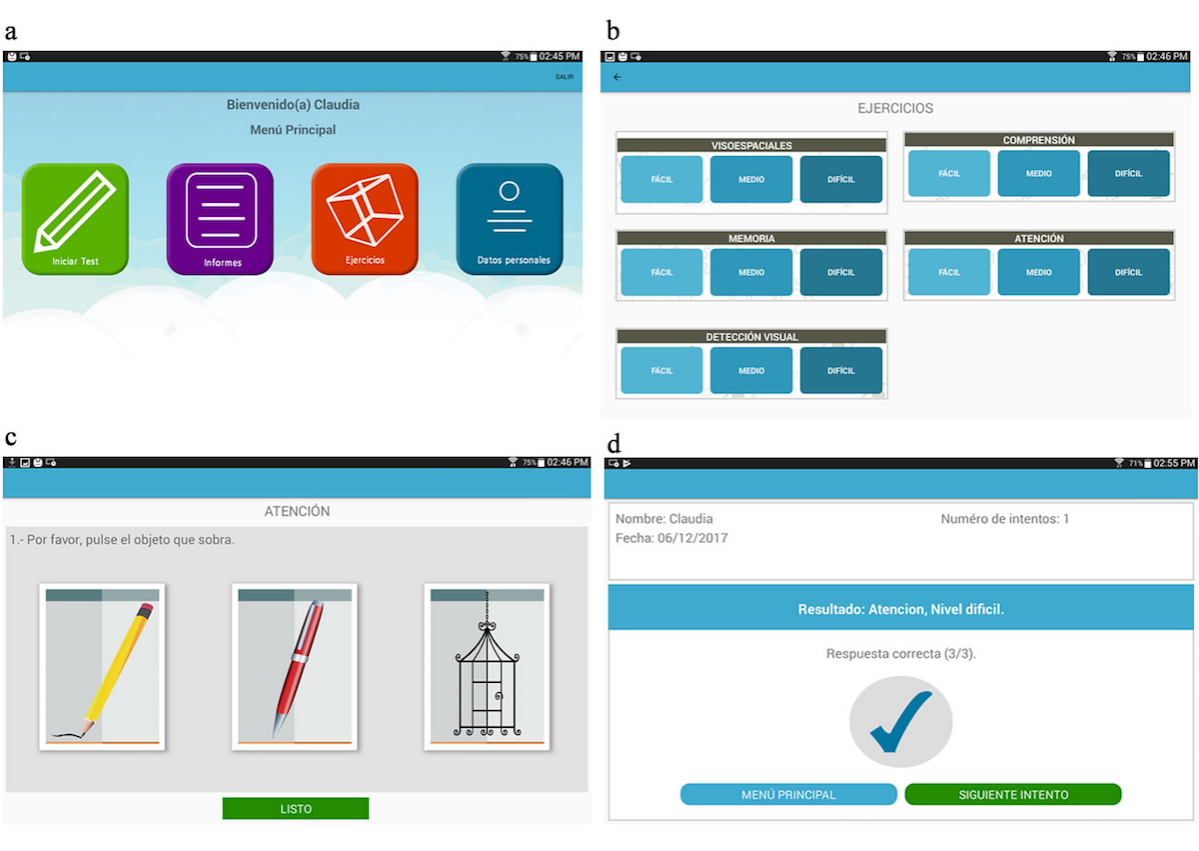
Mobile app screens: (a) main menu, (b) exercise menu, (c) memory exercise level 1, attempt 1, (d) results screen.

A clear, simple, and attractive design was chosen, in which the user was able to access all the exercises and levels directly from the main menu (Figure 1). Additional considerations were taken to simplify the tasks (Textbox 2).

**Table 1:** A comparison between the characteristics of the experimental and control groups in this study.

Characteristic	Experimental group, mean (SD)	Control group, mean (SD)	*t* test	*P* value
Age (years)	67 (7)	71 (5)	1.53	.14
Education level (years)	8 (4)	11 (4)	1.45	.16

**Guidelines used in the design of the iBeni mobile app.**

- The app interfaces presented large-sized elements such as text, icons, images, and buttons.
- The color palette selected for the interface keeps conservative colors and maintains contrast in the foreground and background, especially in text messages.
- The structure on each screen keeps a well-organized distribution of its elements.
- Too complex interface designs should be avoided; hence irrelevant information is minimized on general screens, as well as on screens containing exercises.
- The number of clicks within the app is optimized to keep track of the user’s location in the app.
- Simple tasks that do not require long attention spans are implemented.

**Optimizations considered to simplify participant use of the iBeni mobile app.**

- The main menu is simplified so that the user can define the level of the exercise with just two clicks.
- The display of the exercises shows simple directions that indicate the user what to do.
- Each result screen displays the name of the user, number of attempts, type of exercise, level, date, and number of right answers.
- The use of scrollbars was avoided since this movement seemed challenging for the users.
- All the actions in the app are carried out at just 1 click.

#### Database

The app was programmed based on the Android operating system, and contains several screens with forms. These include registration, desertion, modifications, and consultations, which are sent to databases located in a MySQL server in the cloud. This database is comprised of 4 tables containing information from the app (Textbox 3).

**Figure 2:**
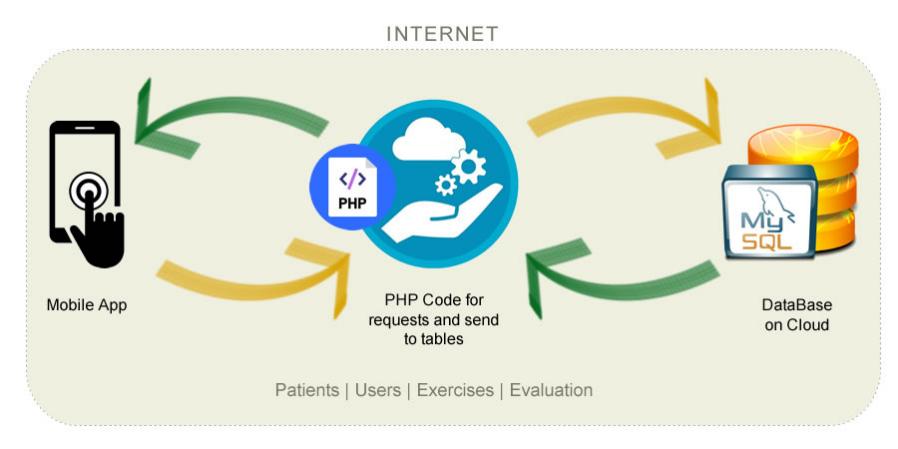
Conceptual representation of the mobile app database.

A hypertext preprocessor (PHP) code was used to store and access the information. The code operates as a Web-based service allowing access and response requests between the mobile device and the database. It works as a bridge so that the Android code generated is capable of interpreting and displaying the information from the database. It also carries out different operations according to the screen on which the user is working (Figure 2).

The mechanism for sending the data entered by the participant from the app to the database occurs in 2 ways. First, for alphanumeric texts, PHP services were adopted, using standardization of characters to ensure the total sending of data. These services have an encrypted connection and security features to safeguard the integrity of the app. Second, for some visual-spatial items, users were due to generate copies of figures and thus these images were protected by a file transfer protocol to guarantee their protection.

The decision to choose the database provider was based on the range of services offered and the low costs involved. However, data integrity was also critical. Recognized companies such as TechRadar and TrustPilot are the best for ease of use, integration of solutions, and security of information. Also, they provide enough storage and database space along with excellent bandwidth.

**The 4 categories that comprise the app database.**

1. Patients: information corresponding to the name, email address, age, sex, marital status, academic level, city and state, and social security affiliation.
2. Users: this information registers the accounts and passwords of patients for their logging into the app.
3. Exercises: it stores dates and times at which an exercise started and concluded, the type of evaluation currently taking place, the level in which it was executed, scores and attempts, the image corresponding to a test and finally the ID and name of the patient.
4. Evaluation: it concentrates the evaluation results corresponding to each type of evaluation executed, the total scores, as well as the date and time at which they were carried out.

### Intervention Sequencing

This was a parallel study. As a nonrandomized trial of a pilot study, the allocation ratio was based on self-allocation. All subjects were summoned to attend the digital workshop, and those who accepted to go to ICSa became the EG with access to the app. In contrast, the CG remained at the IGC facilities. Therefore, no interaction took place between the 2 groups. Nevertheless, care was taken to evaluate how the cognitive performance was before beginning the intervention. This assured that the groups did not statistically differ in age, education, MMSE or Neuropsi assessments. Also, prepilot studies had taken place before the trail began to create and evaluate the design of the app.

Cognitive stimulation among the 2 groups was implemented in 3 months (May-July 2017), from which 12 weeks were devoted to direct intervention. The neuropsychological evaluation was conducted 2 weeks before the start of the program (ie, preevaluation). After the neuropsychological evaluation, the EG received 10-minute training to get familiarized with the interface of the app. It is important to note that both groups carried out the same activities, with the only difference being the methods used. Likewise, the schedule in which they did the activities was identical (ie, 10:00 am-12:00 pm) to ensure that they were conscious and alert during the sessions.

**Figure 3:**
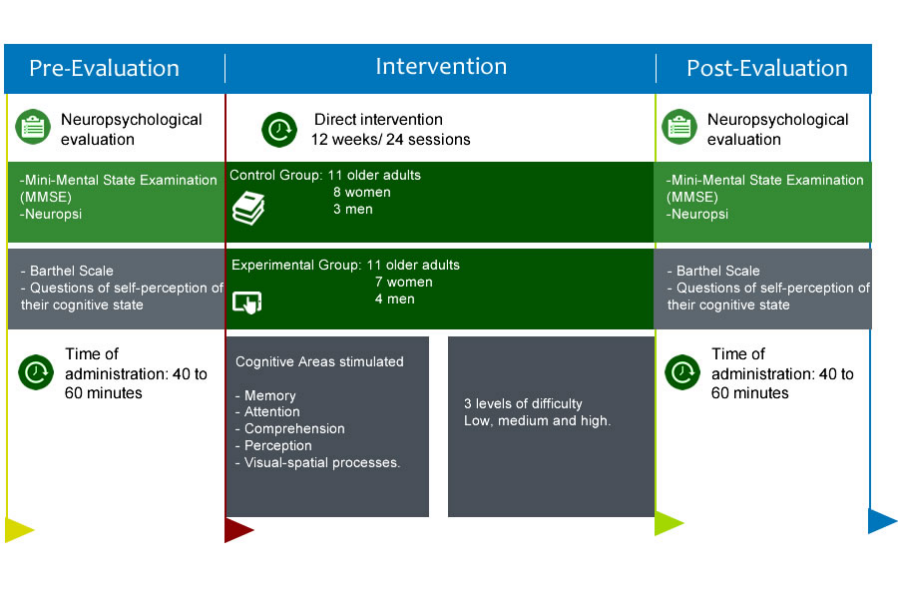
Structure and sequencing of the sessions undertaken by each group.

The cognitive stimulation program consisted of 24 sessions. Participants carried out the exercises assigned according to their progress. At the end of the intervention, all participants were re-evaluated with the neuropsychological and supplementary tests. Figure 3 shows the structure and sequencing of the sessions.

#### Session Procedures

Sessions with the EG group were conducted in the facilities of the computer center at the ICSa. Each participant was provided with a 10.1-inch Samsung Galaxy Tab 4 tablet, in which the app (Version 2017-03-08) was installed and configured. Sessions for the CG group were carried out in a classroom of the IGC de Punta Azul. The materials used consisted of 2 manuals. One was for the examiner, in which answers, partial and total scores, attempts, level and date of the session were registered. The other manual was for the participants, in which the exercises they had to work on were provided. Every week, the stimuli, number of exercises, levels and attempts were made as equal as possible. In the first week, both groups were assigned memory, attention, and perception exercises at a low level of difficulty. As participants progressed, more exercises were added and the level of difficulty gradually increased (Multimedia Appendix 3). Table 2 describes the exercises programmed for the 24 sessions.

**Table 2:** A description of the cognitive stimulation exercises included in the intervention.

Cognitive function	Exercise description	Exercise materials	Number of levels
Memory	These exercises allowed the senior to test information retention capacity in each period. The participants had to observe an image, and then had to respond to related questions. The number of questions increased as the participants progressed from one level to the next.	12 images depicting places, people, and landscapes. Open-ended questions related to the image.	3 levels with 4 attempts
Attention	These exercises aimed to preserve the level of intellectual and association skills in older adults. They consisted of showing a series of similar images, in which they had to point out which was different or was not related to the rest.	68 images	3 levels with 4 attempts
Comprehension	These exercises were aimed at preserving the degree of interpretation and perception in the senior. Each was presented as a series of directions for the participant, and they had to execute them by pointing to and marking the corresponding images. As levels were reached the number and type of geometrical figures were increased.	Series of directions and 72 geometrical figures	3 levels with 4 attempts
Visual detection	The objective of these exercises was to understand whether the older adult discriminates visual stimuli. It consisted of presenting a series of images in which participants must find one or several letters.	768 images of letters	3 levels with 4 attempts
Visual-spatial	They allowed the older adult to mentally represent, transform, and manipulate an object or image. In these exercises, participants had to visualize the image for 40 seconds and then replicate it.	12 abstract images, classified from the number of lines in the figure or from angles. In levels 2 and 3 the participant was given 60 seconds to replicate the image.	3 levels with 4 attempts

## Results

### Overview

All adults lacked experience using ICTs. Only those from the EG attended a digital literacy course before the intervention to develop the necessary technological competencies for efficient app use [38]. Although the purpose of this study was not to promote the level of digital literacy of seniors, it is important to mention that before joining the course the adults had no digital skills. They did not know how to use the computer or the internet. However, after attending the digital literacy course for 4 months at the ICSa facilities, they increased their level so they could carry out the necessary activities on a computer and other electronic devices such as tablets. They also learned to surf the internet and use email.

### Pilot Study

According to the postevaluation in the MMSE, 55% (6/11) of participants from the EG obtained better results in their cognitive skills, and 45% (5/11) maintained their score. The cognitive areas where the results improved were: attention, comprehension, and short-term memory. In the CG, 27% (3/11) of participants obtained better results in the postevaluation, and 64% (7/11) were able to maintain their score. However, 9% (1/11) of seniors obtained a lower score.

Significant values for results obtained from the MMSE in the EG versus the CG are shown in Table 3. A mixed analysis of variance (ANOVA) (2X2) was used to compare the effects of the interventions. Only the repeated measures effect in both groups was significant (*F*_1,22_=4.59, *P*=.04), while there were not differences between groups (*F*_1,20_=0.18, *P*=.67), neither an interaction between them (*F*_1,22_=2.04, *P*=.16). The Cohen *d*=0.45 indicated a minimal effect. The Neuropsi results did not show significant differences. The repeated measures effect in both groups did not differ (*F*_1,22_=1.98, *P*=.17). Also, there were no differences between groups (*F*_1,20_=0.10, *P*=.75), and no interaction between them (*F*_1,22_=1.44, *P*=.24).

**Figure 4:**
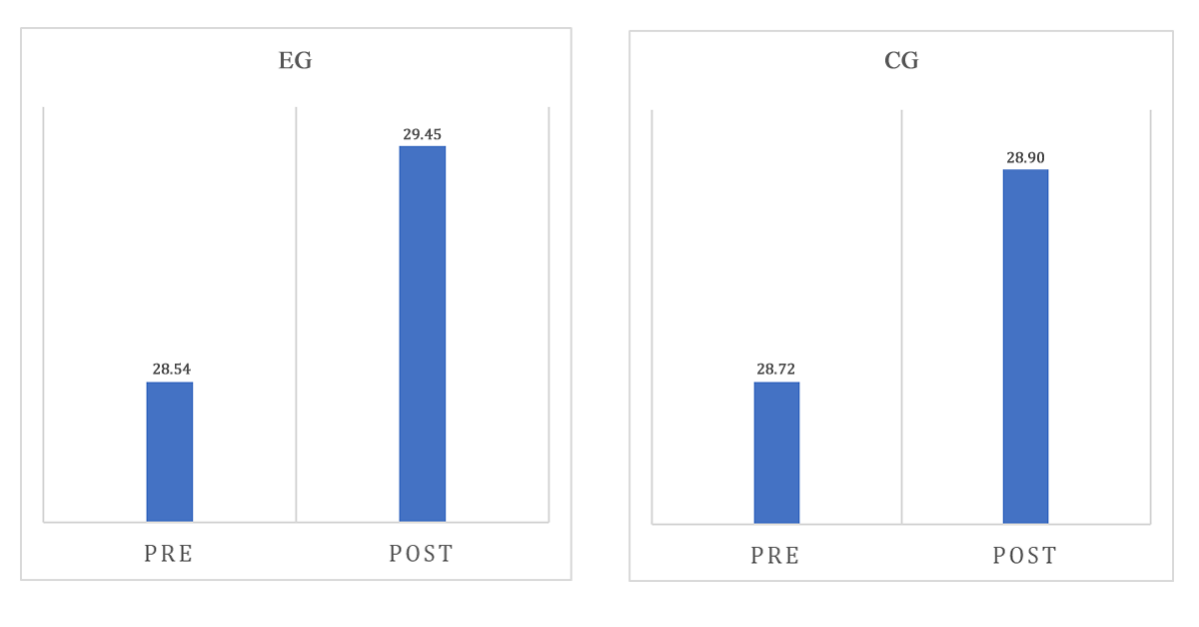
Results of the pre and post evaluation obtained from the Mini-Mental State Examination (MMSE). EG: experimental group; CG: control group.

Based on the ANOVA, the repeated measure factor was significant for the MMSE, and two 90% CI levels were computed considering the MMSE preevaluation mean of both groups and another 90% CI for the postevaluation mean of both groups. For the preevaluation mean, the CI=(28.17≤mu≤29.10) and for the mean belonging to the postevaluation, 90 % CI=(28.84≤mu≤29.51; see Figure 4).

**Table 3:** Means and standard deviations of the Mini-Mental State Examination and the Neuropsi test in the pre- and postevaluations in the experimental group compared to the control group.

Neuropsychological test	Experimental group, mean (SD)		Control group, mean (SD)	
	Preevaluation	Postevaluation	Preevaluation	Postevaluation
MMSE^a^	29 (1)	29 (1)	29 (1)	29 (1)
Neuropsi	102 (6)	103 (6)	97 (11)	97 (11)

^a^MMSE: Mini-Mental State Examination.

Concerning autonomy and capacity to perform basic ADL, 100% of the adults maintained their levels of autonomy in the postevaluation according to the Barthel Scale. Regarding questions about cognitive state perception, 73% (8/11) of seniors from the EG reported improved cognitive capacity after the intervention, while 27% (3/11) revealed that they maintained their cognitive state. In the CG, 45% (5/11) of older adults perceived an improvement in their cognitive capacity, while 55% (6/11) indicated that their memory and cognitive functions remained unchanged after the intervention.

### Validation of the Mobile Application

With the aim of adequately evaluating the relevance of the app, it was essential that users analyzed its usefulness, performance, and design by providing feedback on existing failures, any concrete benefits, and areas for improvement. After the intervention in both groups, the EG was invited to evaluate the acceptability and viability of the app. For this validation, a technology acceptance test with 17 closed questions was performed based on the technology acceptance model (TAM). This test has been widely used to predict the acceptance of new technologies. Also, TAM has been built on collective findings suggesting that the desired technology was substantially dependent on user acceptance [39,40].

To understand the benefits from the use of this kind of medium, interviews were conducted to explore any concerns, reasons, and contexts related to the decision of using or not using this type of app. The interviews with the seniors also allowed for obtaining detailed answers. The tests, as well as the interviews, were performed between July and August 2017. The parameters used for the validation of the app were: (1) ease of use, (2) functionality, (3) design, (4) usefulness, and (5) satisfaction. In this study, the ease of use of the app was defined as the degree to which the user believes that using the app will be effortless. Meanwhile, the usefulness of the app is defined as the degree to which the user believes that using the app would enhance his or her cognitive state. The TAM posits that the usefulness and ease of use perceived have a direct effect on attitudes and satisfaction using new technology. The parameters of the functionality and design are the degree to which the user believes that the functionality and design quality of the app is correct. The measurement used in the questionnaire corresponds to a 5-point Likert scale. The options in the scale appeared as follows: strongly agree (5), agree (4), neutral (3), disagree (2), and strongly disagree (1).

Concerning the analysis of the first parameter, *ease of use*, 91% (10/11) of EG participants agreed to a positive evaluation, confirming that the app is easy to use in both the evaluation and the exercise sections. Thus, interaction with the interface is clear and intuitive. Regarding *perceived usefulness*, all the participants had a favorable agreement, suggesting that the app allowed them to keep their mind active and that this kind of app is helpful to this type of population. Moreover, when the possibility of using an app for cognitive stimulation was presented to the older adults, they showed a positive attitude towards the idea. During the evaluation of the third parameter, *Attitude towards the Use of Technology*, all the participants maintained a positive attitude, which suggests that the use of apps for cognitive stimulation is a valuable project. Similarly, participants indicated that this type of technology allows cognitive stimulation to be efficient, it is easy to perform, and, above all, it has an attractive design. In the parameter *Intention of Use*, 82% (9/11) of the participants indicated that they would use the app and other cognitive activation technologies as much as they can. This shows that there is a need to create similar apps that are more accessible for this population. Only 9% (1/11) of participants was neutral regarding the use of technological methods for cognitive activation. Lastly, the parameter *Satisfaction* received a favorable evaluation from all the participants, meaning that the app provides an attractive interface to improve cognitive performance in older adults and to decelerate cognitive impairment processes. Table 4 shows the raw marks obtained for each parameter.

Regarding the participant interviews, the responses obtained suggested that this type of app offers several opportunities. During an interview, the older adults were asked in an open question format what benefits they perceived when using the app. Some of the benefits highlighted were: visualization of results in real time, customizable interface, automatic registration, more active cognitive stimulation, and time-saving. Visualization of the results was the most commonly mentioned benefit. As one senior explained:

For me it is important to visualize the results that I got in the exercises I did, because many times these data are not provided to us.

EG participant, female

The participants also observed that the app allowed them to have a more active and personalized stimulation. Additionally, the seniors noticed that during intervention many tasks were not adequate for traditional paper execution because they required the presence of various stimuli or the inhibition or delayed presentation of specific elements. Consequently, the app allowed broader interaction, which was fundamental for the accomplishment of some tasks.

**Table 4:** The technology acceptance model validation results for the iBeni mobile app.

Parameter	Mean (SD)
Participant ease of use	7 (1)
Perceived usefulness	7 (0)
Attitude towards using of technology	7 (1)
Intention of use	7 (1)
Satisfaction	7 (1)

Also, about the perceived benefits, participants were asked whether they would be willing to use the app in the future. Every participant stated that they would be willing to use the app for cognitive stimulation exercises. Finally, it was clear that while the seniors recognized the app’s benefits, it should be used as a supplement in health care. However, it must not be used to replace the human contact. As one older adult said:

Well, I think people contact is very important, and I wouldn’t want the app to be used excessively.

EG participant, female

In other words, health care systems should supplement, but not replace, health care professionals or the attention given by family members.

## Discussion

### Principal Results

It is a fact that adults over 60 years of age express their concern regarding the decline in their mental skills. Several studies assert that change in cognitive skills and mental processes is related to the aging process and the person’s quality of life. However, there are very few studies of older Mexicans adults that have discussed whether the improvement of cognitive functions can have short or long-term effects with the use of technology. The studies mentioned above have found that cognitive interventions based on ICT can result in improvements in many perceptual and cognitive abilities [27-35]. Thus, these types of interventions are potentially an ideal solution to address the many perceptual and cognitive declines associated with aging. The study by Pereira-Morales et al [35] suggested that cognitive training of moderate intensity, supported by a web platform, could lead to significant improvements in cognitive and psychological well-being in older people with subjective memory complaints. Shellington et al [30] argue that sixty percent found the app was easy to use or similar to what they experienced with square-stepping exercise in the laboratory setting. Most said they would continue to use the HealtheBrain app and would recommend it to friends and family. Chan et al [29] stated that the results yielded evidence for more significant improvement over time in the iPad intervention compared with the control groups for processing speed and episodic memory. Thus, the program was successful at improving cognitive performances through productive engagement and provided an added benefit of technological mastery. Finally, Yasini and Marchand [28] indicate that the use of tablets and the structure of serious games in close cooperation with health professionals and elderly patients are likely to provide satisfactory results to improve health care provided for elderly patients suffering from cognitive disorders.

According to the analysis carried out after the cognitive intervention in this study, Both EG and CG obtained better results in the neuropsychological evaluation, even when the CG could not execute the exercises repetitively. Concerning the adult who obtained a low mark in the postevaluation he is suspected to have been distracted during the evaluation. As to the validation of the app, it was found that most of the participants showed a positive attitude regarding its use. This indicates that it is easy to use, accessible and that it allows cognitive stimulation to be efficient and friendly. Also, the adults indicated that the app provides an attractive interface, which improves cognitive performance and slows cognitive deterioration processes. Some apps for older adults are focused on cognitive training or cognitive assessment. Health care professionals use other apps only for interpretation and presentation of results. The app in this study combines training and presents the results for all users. Likewise, the exercises included in the app were specially designed for the elderly population and were based on standards and validated tests, such as the MMSE and the Neuropsi. It is important to highlight this point because it allowed seniors to have a better perception of instructions, images, and interaction with the app. This is unlike other apps created to suit the specific needs of only the physician. Finally, in Mexico, there is no documented evidence of a similar app for the older adult. This is the first pilot study on this topic in the country. Lastly, participants perceived this digital support as more attractive and ludic, resulting in higher motivation and emotional levels. An element that played an essential role in the participants’ acceptance of the technology was the fact that the EG participated in a Digital Literacy Workshop before implementation. This might have generated greater confidence when executing exercises on the tablet.

### Limitations

There are many limitations to this study. They are mainly related to the number of adults who took part in the intervention. This sample is a nonrepresentative one, and underrepresentation of the whole population could have taken place. Nevertheless, we followed the World Medical Association Declaration of Helsinki [41] on the C30 principle, and every subject had access to the diagnostic and therapeutic methods. Even though it is necessary to consider that very few interventions of this kind have been implemented and documented in Mexico, this pilot study may be used to gain funding for a larger, more thorough research project. A more systematic study and with a broader scope is required to determine if the benefits found in this pilot study could be replicated. Randomized trials in subsequent studies may prove this type of treatment efficacious.

### Conclusions

This article describes a pilot study and the validation of a cognitive stimulation app for adults over 60 years of age. The technical developments considered for the design of the app emphasize the importance of adopting user-centered design methodologies, especially for the development of apps aimed at the elderly population. The results of this study highlight that both groups obtained better results in the postevaluation. The participants were able to execute the exercises repetitively, which the CG could not accomplish since they had to respond on the manual and no further attempts were provided. However, both groups increased their score in the neuropsychological evaluations. Even when the MMSE and the Neuropsi evaluate almost the same psychological functions, the Neuropsi considers 130 as its maximum score, versus the 30 items of the MMSE. Therefore, a general improvement in both groups seems to have taken place.

Another important point is that the exercises were not the same as the tests, and besides levels of difficulty were added, even when a learning effect might have been taken place in both groups. It was possible to demonstrate that the stimulation provided by using an app is appropriate and that it allows adults to stimulate or maintain their cognitive capacity. This was reflected in better results and the preservation of cognitive capacity.

Validation of the app emphasizes the perceived benefits and the relevance of adopting ICTs by older adults, especially for their health care. In the validation analysis, all the participants were aware of the convenience of ICTs for cognitive stimulation. Since the 22 participants reported to have the intention of using technological methods to improve their cognitive skills, it is clear that there is a specific need for creating more services like this one.

Finally, it must be recognized that to carry on this study, technical and institutional requirements had to be fulfilled. Older adults must have access to this technology to gain access to mobile cognitive stimulations due to institutional agreements and permissions. The purchase of ICT devices had to be done because some older adults could not afford their cost. It has been demonstrated that interventions are capable of improving cognition, but the importance of their structure and deliverance to ensure that people become engaged must also be considered.

For future work, more exercises are being designed to be integrated into the app. These exercises seek to stimulate other cognitive areas that were not included in this study. We will continue with the commitment of digital literacy of elderly adults from the state of Hidalgo, Mexico. This will help the elderly adults to develop the necessary technical skills that allow them to use the app more efficiently. Also, the authors consider using a “waiting group” in the future to compare this functioning.

## Appendix

Multimedia Appendix 1 Cognitive interventions based on Information and Communication Technologies (ICT) reported by type of intervention, population, country, cognitive functions and significant findings.

Multimedia Appendix 2 Informed consent.

Multimedia Appendix 3 Description of the cognitive stimulation exercises included in the intervention.

## Abbreviations

ADL: activities of daily living
ANOVA: analysis of variance
CG: control group
EG: experimental group
IGC: Centro Gerontológico Integral of Pachuca de Soto
ICT: Information and Communication Technologies
ICSa: Instituto de Ciencias de la Salud
MCI: mild cognitive impairment
MMSE: Mini-Mental State Examination
PHP: hypertext preprocessor
UAEH: Universidad Autónoma del Estado de Hidalgo
TAM: Technology Acceptance Model
