# Supplementary material for: A Mobile App (iBeni) With a Neuropsychological Basis for Cognitive Stimulation for Elderly Adults: Pilot and Validation Study
Source: JMIR Res Protoc. 2018 Aug 21;7(8):e172. doi: 10.2196/resprot.9603 (PMC6123536; doi:10.2196/resprot.9603)
Supplement: Multimedia Appendix 1 [file resprot_v7i8e172_app1.pdf]

Table 1. Cognitive Interventions based on ICT Reported by Type of intervention, N, Country, Cognitive Functions and Significant Findings

Study	Type of intervention	Duration	N	Country	Cognitive Functions Evaluated	Significant Findings
Walton, et. al. 2015 [27]	Cognitive training; active control group	28 days	28 healthy older adults (mead age 67.4)	Australia	Visual, and executive functions, memory, Arithmetic, attention	Improved performance in multiple measures of processing speed; visual working memory can be enhanced over a short period of computarized cognitive training.
Zygouris, et. al. 2015 [28]	Cognitive assessment: Application: Virtual Supermarket	Not specified	55 older subjects, 21 healthy ol der adults and 34 older subjects with MCI	Greece	Not specified	The virtual supermarket appears to be a valid method of screening for MCI in an older adult population though it cannot be used for mild cognitive impairment subtype assessment.
Zorluoglu, et. al. 2015 [29]	Cognitive assessment: Mobile cognitive screening (MCS); active control group.	Not specified	23 older subjects with MCI (mean age 81.78)	Turkey	Arithmetic, orientation, abstraction, attention, memory, language, visual, and executive functions	The proposed test was able to differentiate the individuals in the control and dementia groups for executive, visual, memory, attention, orientation functions with statistical significance.
Yasini, et. al. 2016 [30]	Cognitive Stimulation: Mobile Health	6 moths	15 older subjects	France	Memory, attention, concentration	The results are promising and can pave the way for improving

	Application, Stim'Art.					cognitive function in the elderly patients. The use of tablets and the constitution of serious games in close cooperation with health professionals and elderly patients (the end user), are likely to provide satisfactory results to improve healthcare provided for elderly patients suffering from cognitive disorders.
Chang, et. al. 2016 [31]	Cognitive training: The iPad intervention program; active control groups.	10 weeks	11 older subjects (age 60-90)	USA	Processing speed<br>Mental control<br>Episodic memory<br>Visuospatial processing	The results yielded evidence for greater improvement over time in the iPad intervention compared with the control groups for processing speed and episodic memory.<br>Thus, the program was successful at improving cognitive performances through productive engagement and provided an added benefit of technological mastery.
Lu, et. al. 2017 [32]	Cognitive training games: Mobile game	Not specified	9 older subjects (age 82-90)	Taiwan	Attention<br>Executive function<br>Memory<br>Lenguaje<br>Visuospatial function	The results showed that the cognitive training game developed in this study was accepted by the participants, and a high degree of satisfaction was noted. Moreover, the elements of the interface, including its size, layout and control flow, were tested and found to be suitable for use.

Shellington, et. al. 2017 [33]	Cognitive Stimulation: Mobile application: HealtheBrain app.	3 weeks	19 older subjects (mean age 68.3)	Canada	Memory by walking simple patterns at home or anywhere	Sixty percent found the app was easy to use or similar to what they experienced with square-stepping exercise in the laboratory setting. Most said they would continue to use the Health*e*Brain app and would recommend it to friends and family. The authors believe that their findings in a representative cohort support the HealtheBrain app as a scalable intervention to promote cognitive health in older adults.
Han, et. al. 2017 [34]	Cognitive training Tablet-based Spaced retrieval training program: USMART.	4 weeks	20 older subjects with MCI	Korea	Word List Memory Test<br>Word List Recall Test<br>Word List Recognition Test.	The USMART group had larger improvements in Word List Recall Test score than the usual care group. There were no significant differences in other primary or secondary measures between the USMART and usual care groups. Moreover, no USMART-related adverse events were reported.
Pereira-Morales, et. al. 2018 [35]	Cognitive training Web platform: Cognitive training progra m; active control group.	8 weeks	12 older subjects with subjective memory complaint s (mean age 66.4)	Colombia	Psychostimulation program	This study suggested that cognitive training of moderate intensity, supported by a web platform, could lead to significant improvements in cognitive and psychological well-being in older people with subjective memory complaints.
