# Supplementary material for: A Mobile App (iBeni) With a Neuropsychological Basis for Cognitive Stimulation for Elderly Adults: Pilot and Validation Study
Source: JMIR Res Protoc. 2018 Aug 21;7(8):e172. doi: 10.2196/resprot.9603 (PMC6123536; doi:10.2196/resprot.9603)
Supplement: Multimedia Appendix 2 [file resprot_v7i8e172_app2.pdf]

Informed consent

iBeni mobile application aims at improving cognitive functions and at slowing the progression of cognitive decline in healthy older adults or in older adults with mild indicators of decline in some cognitive areas.

The cognitive areas to be stimulated by *iBeni* are: memory, attention, comprehension, perception and visuospatial processes. Each of the exercises will include three levels of difficulty (low, medium and high), which will be enabled as you progress.

Before starting the exercises, an initial neuropsychologically-based assessment will be carried out, which will allow us to know your cognitive state. The initial assessment lasts 40 minutes approximately. This assessment will be applied again after the intervention.

During the intervention weeks, those responsible for the project (the authors) will indicate you the type of exercises and the level you should realise. As you progress, more exercises will be added and the difficulty level will gradually increase.

If you give your consent to participate in this study, You accept that your participation will be voluntary, without coercion or force. We also understand that you have the right to end your participation whenever you wish to do so.

Likewise, the responsible entities will take the necessary measures to ensure the confidentiality of all the information you provide, guaranteeing that your data will not be revealed under any circumstance.

**I agree to take part in the study**	**I don´t agree to take part in the study**

Full name and signature

Carretera Exhacienda La Concepción S/N San Juan Tilcuautla, San Agustín Tlaxiaca, Hgo; C.P. 42160
Teléfono 01 (771) 717-2000 Ext. 4314 y 4315

Carretera Exhacienda La Concepción S/N San
Juan Tilcuautla, San Agustín Tlaxiaca, Hgo; C.P.
42160
Teléfono 01 (771) 717-2000 Ext. 4314 y 4315
