# Supplementary material for: A Mobile App (iBeni) With a Neuropsychological Basis for Cognitive Stimulation for Elderly Adults: Pilot and Validation Study
Source: JMIR Res Protoc. 2018 Aug 21;7(8):e172. doi: 10.2196/resprot.9603 (PMC6123536; doi:10.2196/resprot.9603)
Supplement: Multimedia Appendix 3 [file resprot_v7i8e172_app3.pdf]

Table 2. Description of the cognitive stimulation exercises included in the intervention

Area

Description

Materials

Number of Levels

Memory

These series of exercises allows the older adult to test information retention capacity in a given period of time.

The participants have to observe an image attentively and subsequently they have to respond to related questions. As participants progressed from one level to the next, the number of questions increases.

12 images depicting places, people, landscapes. Open-ended questions related to the image.

3 levels with 4 attempts

Attention

The aim of these exercises is to preserve the level of intellectual and association skills in older adults. They consist in showing them a series of similar images, in which they have to point out which image is different or is not related to the rest.

68 images

3 levels with 4 attempts

Comprehension

These exercises aim at preserving the degree of interpretation and perception in the older adult. They are presented as a series of directions for the participant and they have to execute them pointing to and/or marking the corresponding images. As levels are accomplished, the number and the type of geometrical figures are increased.

Series of directions

72 geometrical figures

3 levels, 4 attempts

Visual Detection

The objective of these exercises is to know whether the older adult presents discriminate visual stimuli. It consists in presenting a series of images in which participants must find one or several letters.

768 images of letters

3 levels with 4 attempts

Visual-spatial

They allow the older adult to mentally represent, transform and manipulate an object or an image. In these exercises, participants have to visualize the image during 40 seconds and subsequently they have to replicate it.

12 abstract images, classified from the number of lines in the figure or from angles. In levels 2 and 3 the participant is given 60 seconds to replicate the image.

3 levels with 4 attempts
